# Indirect Band Gap in Scrolled MoS_2_ Monolayers

**DOI:** 10.3390/nano12193353

**Published:** 2022-09-26

**Authors:** Jeonghyeon Na, Changyeon Park, Chang Hoi Lee, Won Ryeol Choi, Sooho Choi, Jae-Ung Lee, Woochul Yang, Hyeonsik Cheong, Eleanor E. B. Campbell, Sung Ho Jhang

**Affiliations:** 1 School of Physics, Konkuk University, Seoul 05029, Korea; 2 Center for Integrated Nanostructure Physics, Institute for Basic Science, Suwon 16419, Korea; 3 Department of Physics, Ajou University, Suwon 16499, Korea; 4 Department of Physics, Dongguk University, Seoul 04620, Korea; 5 Department of Physics, Sogang University, Seoul 04107, Korea; 6 EaStCHEM, School of Chemistry, Edinburgh University, David Brewster Road, Edinburgh EH9 3FJ, UK; 7 Department of Physics, Ehwa Womans University, Seoul 03760, Korea

**Keywords:** rolled structure, 1D structure, MoS_2_, scrolled MoS_2_, band gap, ionic liquid gating

## Abstract

MoS_2_ nanoscrolls that have inner core radii of ∼250 nm are generated from MoS_2_ monolayers, and the optical and transport band gaps of the nanoscrolls are investigated. Photoluminescence spectroscopy reveals that a MoS_2_ monolayer, originally a direct gap semiconductor (∼1.85 eV (optical)), changes into an indirect gap semiconductor (∼1.6 eV) upon scrolling. The size of the indirect gap for the MoS_2_ nanoscroll is larger than that of a MoS_2_ bilayer (∼1.54 eV), implying a weaker interlayer interaction between concentric layers of the MoS_2_ nanoscroll compared to Bernal-stacked MoS_2_ few-layers. Transport measurements on MoS_2_ nanoscrolls incorporated into ambipolar ionic-liquid-gated transistors yielded a band gap of ∼1.9 eV. The difference between the transport and optical gaps indicates an exciton binding energy of 0.3 eV for the MoS_2_ nanoscrolls. The rolling up of 2D atomic layers into nanoscrolls introduces a new type of quasi-1D nanostructure and provides another way to modify the band gap of 2D materials.

## 1. Introduction

Two-dimensional (2D) atomic layers of graphene and transition metal dichalcogenides (TMDs) have been widely investigated for future applications [1]. Among TMDs, materials having semiconductor properties, such as MoS_2_ and WS_2_, have received a great amount of attention as materials that complement gapless graphene. The structural modifications of 2D materials, such as ripples, and folded and rolled structures, were also studied [2,3,4]. Since a rolled structure has a large surface area in a small volume and can transport materials through the hollow core, the nanoscrolls of 2D materials have potential for energy storage, sensors, microrockets, and photodetectors [5]. The rolling up of graphene [4,6,7,8], h-BN [9], and TMDs into nanoscrolls [10,11,12,13,14,15,16,17,18,19,20,21] was achieved, and the properties of nanoscrolls are of interest from theoretical and experimental viewpoints. In particular, nanoscrolls spirally wrapped from 2D sheets provide another possibility to tune the band gap of 2D materials. Theory predicts band structure changes in graphene nanoscrolls [22] and black phosphorus [23]. Chirality and radius are the most relevant factors to determine the electronic structure of nanoscrolls. One can also infer from previous studies on MoS_2_ nanotubes [24] where the band gap decreased with decreasing diameter that a change in the band gap of MoS_2_ can be expected upon scrolling. However, experimental studies of the band gap of MoS_2_ nanoscrolls often reported contradictory results. Metallic transport with a zero band gap was claimed for a scrolled MoS_2_ [13], and a small red shift of 30–60 meV in the A peak (direct band gap) compared to a MoS_2_ monolayer was reported from the photoluminescence (PL) spectroscopy of MoS_2_ nanoscrolls [10,16].

In this paper, we produced MoS_2_ nanoscrolls that had inner core radii of ∼250 nm by rolling up MoS_2_ monolayers, and investigate their optical and transport band gaps using PL and ambipolar transport in ionic-liquid-gated transistors. Combined with atomic force microscopy (AFM) to determine the structures of the nanoscrolls complemented with Raman spectroscopy, we provide a comprehensive characterization of the properties of MoS_2_ nanoscrolls and verify the possibility to tune the band gap compared to the MoS_2_ monolayer.

## 2. Materials and Methods

Experiments were performed on MoS_2_ monolayers prepared with either chemical vapor deposition (CVD) or mechanical exfoliation. CVD was carried out in a two-zone quartz tube furnace with sulfur powder (∼1 g) loaded into a low-temperature *T* zone heated to 400 °C and MoO_3_ film (∼50 nm) into a high-*T* zone heated to 800 °C. The CVD growth produced triangular crystals (ca. 25 μm edges) of MoS_2_ on SiO_2_ substrates. Exfoliated MoS_2_, on the other hand, was obtained with a gold-assisted exfoliation technique [25] that yields monolayers with much larger lateral dimensions than ∼10 μm.

The method used to produce scrolls from monolayers follows the one originally introduced by Xie et al. [4]. A droplet of isopropyl alcohol (IPA) solution (IPA:deionized water volume ratio of 1:3) was placed on top of MoS_2_ on the substrate. For exfoliated MoS_2_, a 1 mM KOH in IPA solution was used to facilitate rolling up the monolayer.

Raman and PL measurements were conducted using a home-built confocal microscope system. A 532 nm (2.33 eV) beam of a diode-pumped solid-state (DPSS) laser was used as an excitation source. The scattered or emitted light was dispersed with a Jobin-Yvon Horiba iHR550 spectrometer (2400 grooves/mm for Raman and 300 grooves/mm for PL) and detected with a liquid-nitrogen-cooled back-illuminated charge-coupled-device (CCD) detector. In order to access the low-frequency range down to 5 cm^−1^, volume holographic filters (OptiGrate Corp, Oviedo, FL, USA) were used to reject the Rayleigh-scattered light. Laser power was kept below 100 μW (focal spot ∼1 μm^2^) to avoid damaging the samples. The PL spectrum of the scrolled MoS_2_ was fitted using the multipeak Lorentzian fitting analysis of OriginPro 2016 software (Northampton, MA, USA) by setting the initial positions of A series (A${}_{xx}$, A${}^{-}$, A) and B exciton peaks to the known positions for a monolayer MoS_2_ [26].

Transistors incorporating rolled-MoS_2_ were fabricated on SiO_2_ (300 nm)/Si substrates. The source and drain electrodes were patterned with conventional electron-beam lithography. Ti (20 nm)/Au (100 nm) electrodes were then deposited for transistors with backgating, and Cr (30 nm)/Au (120 nm) for transistors with ionic liquid-gating to avoid the strong electrochemical reaction of Ti. Diethylmethyl(2-methoxyethyl)ammonium bis(trifluoromethylsulfonyl)imide (DEME-TFSI) was used as the ionic liquid. Ionic liquids have hygroscopicity, and moisture penetrates easily. Moisture penetration lowers the charge density of the ionic liquid and provides a channel for leakage current. To minimize this effect, the device was heated in a low vacuum for 12 hours at 370 K and then cooled for 2 hours at 200 K. Transistor characteristics with ionic liquid gating were then studied at 250 K using a vacuum probe station and a Keithley 4200 semiconductor characterization system in the core facility center for quantum characterization/analysis of two-dimensional materials and heterostructures. Backgated transistors were investigated using a Quantum Design PPMS that could reach temperatures down to 2 K.

## 3. Results and Discussion

### 3.1. Structure of Scrolled MoS_2_

**Figure 1:**
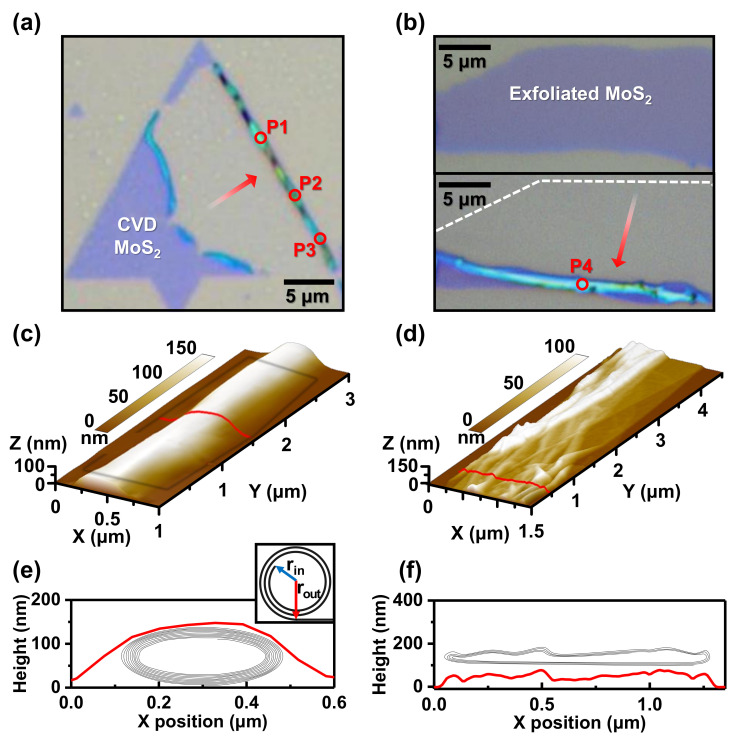
(**a**) Optical image of a nanoscroll produced from CVD-grown MoS${}_{2}$. (**b**) Optical image of an exfoliated MoS${}_{2}$ before (top) and after scrolling (bottom). (**c**,**d**) AFM 3D images of each nanoscroll in (**a**,**b**). (**e**,**f**) Schematic diagrams of internal structures and cross-sectional profiles of each following the red line shown in the AFM 3D images. (**e**) Inset shows an ideal circular scroll with an Archimedean spiral structure.

In total, 15 MoS_2_ scrolls were produced with droplets of IPA solution, 2 of which were from CVD-grown MoS_2_ and 13 from exfoliated monolayers. Figure 1a,b present optical images of typical scrolls produced from CVD-grown MoS_2_ and exfoliated monolayer, respectively. Figure 1a shows MoS_2_ scrolls from the center towards the edge of a triangular MoS_2_ crystal. This suggests that the orientation of the nanoscroll was parallel to the edge and to the armchair direction, consistent with a previous report [13]. The formation energy for rolled-up MoS_2_ scrolling in armchair orientation is lower than that for the zigzag and other chiralities [13]. MoS_2_ nanoscrolls were studied on samples prepared from CVD-grown MoS_2_ [10,13,14,15,16]; here, we also conducted research with scrolls prepared from exfoliated MoS_2_. CVD-grown MoS_2_ on a SiO_2_ substrate often possesses strain as it is synthesized at high temperature and its thermal expansion coefficient is ∼1000 times larger than that of the SiO_2_ substrate [11]. Due to the built-in strain, CVD-grown MoS_2_ is easily separated from the substrate and scrolled using the IPA solution. For exfoliated MoS_2_, however, a small amount of KOH must be added to the IPA solution to help in the separation of 2D sheet from the substrate by etching SiO_2_. Figure 1b shows an optical image of an exfoliated MoS_2_ before scrolling (top), and an optical image of a MoS_2_ nanoscroll after rolling up with a droplet of IPA + KOH solution (bottom). The nanoscroll was rolled about 10 μm from the upper edge of the 2D sheet along the direction of the red arrow shown in Figure 1b.

The structure of the MoS_2_ nanoscrolls was studied with atomic force microscopy (AFM) for all 15 nanoscrolls. Three-dimensional AFM images are displayed in Figure 1c,d for the nanoscrolls shown in Figure 1a,b with the cross-sectional profiles of each in Figure 1e,f. As reported for rolled-up graphene [6], the scrolls are distorted from the ideal circular–cylindrical form (inset of Figure 1e). The MoS_2_ nanoscroll shown in Figure 1c had an elliptical–cylindrical structure in the cross-section due to the interaction with the underlying substrate (Figure 1e), with a height (∼150 nm) to width (∼400 nm) ratio of ∼0.4. On the other hand, the MoS_2_ nanoscroll displayed in Figure 1d shows a collapsed ribbonlike structure. The height along this nanoscroll was nonuniform, with an average height (∼60 nm) to width (∼1300 nm) ratio of ∼0.05. Among the 15 investigated nanoscrolls, 3 showed an elliptical structure with a height/width ratio larger than 0.1, 7 had a collapsed ribbonlike structure, and the others showed different shapes (such as a partially collapsed structure) that did not fall exactly into these two categories (elliptical or collapsed).

The number of stacking layers in nanoscrolls can be estimated by considering both the dimension of the MoS_2_ prior to scrolling and the cross-sectional profile of the nanoscroll after scrolling [6]. Using the interlayer spacing of MoS_2_ nanoscrolls t≃ 0.65 nm [10], the MoS_2_ nanoscroll in Figure 1a,c was estimated to consist of MoS_2_ that was rolled for ∼10 turns. By comparing the height of 10 stacked MoS_2_ rolls (2×0.65 nm ×10 layers = 13 nm) to the height of the elliptical MoS_2_ nanoscroll (∼150 nm), it is reasonable to assume that a large hollow core was present in the nanoscroll. For the collapsed MoS_2_ nanoscroll shown in Figure 1b,d, the height of the estimated number of stacking layers (4 layers giving 5.2 nm) was much smaller than the height of the collapsed scroll (∼60 nm), implying the presence of a hollow core within the collapsed scroll. Schematic illustrations of the internal structures of the MoS_2_ nanoscrolls are given in Figure 1e,f.

To compare hollow core sizes for different shapes of scrolls, we considered the scrolls to have the form of an Archimedean spiral in the inset of Figure 1e, and regarded the innermost radius $r_{\mathrm{in}}$ as the hollow core radius. Following the approach in [6], we obtained $r_{\mathrm{in}}≃140$ and 410 nm for the MoS_2_ nanoscrolls in Figure 1c,d, respectively. The average $r_{\mathrm{in}}$ of all 15 MoS_2_ nanoscrolls was ≃260 nm. Estimated hollow core radii of collapsed ribbonlike structures tend to be larger than those of elliptical structures, with a critical radius for collapse at around $r_{\mathrm{in}}≃$ 250 nm. Carbon nanoscrolls [6] and carbon nanotubes [27] also collapse as the radius of the hollow core increases.

Nanoscrolls theoretically have a radius that minimizes the sum of elastic and surface energies of the system. The following relation shows how the surface energy per unit area γ, bending stiffness *D*, and the length of 2D sheet *B* influence the $r_{\mathrm{in}}$ of the nanoscroll [28]

(1) $$\frac{2\gamma t}{D}=\frac{1}{r_{\mathrm{in}}}-\frac{1}{\sqrt{\left(Bt/\pi \right)+r_{\mathrm{in}}^{2}}}$$

With γ≃400 mJ/m${}^{2}$, D≃1.6×10${}^{-18}$ J for MoS${}_{2}$ [29], and B≃5 μm, the calculation leads to $r_{\mathrm{in}}≃3$ nm. This value is much smaller than the average $r_{\mathrm{in}}≃260$ nm from our experiments or $r_{\mathrm{in}}≃80$–350 nm reported so far from MoS${}_{2}$ nanoscrolls prepared with organic solvents [13,14,15,16]. However, recent studies determined the effective bending stiffness of 2D monolayers in aqueous solution to be approximately three orders of magnitude higher than the value in vacuum [30,31] due to thermal fluctuations and static ripples. Assuming an effective bending stiffness of ≃10${}^{-15}$ J in water/IPA, i.e., 1000 times larger than D≃ 10${}^{-18}$ J for MoS${}_{2}$ in vacuum, Equation (1) results in $r_{\mathrm{in}}\geq 100$ nm, which is consistent with our observation.

### 3.2. Band Gap of Scrolled MoS${}_{2}$

**Figure 2:**
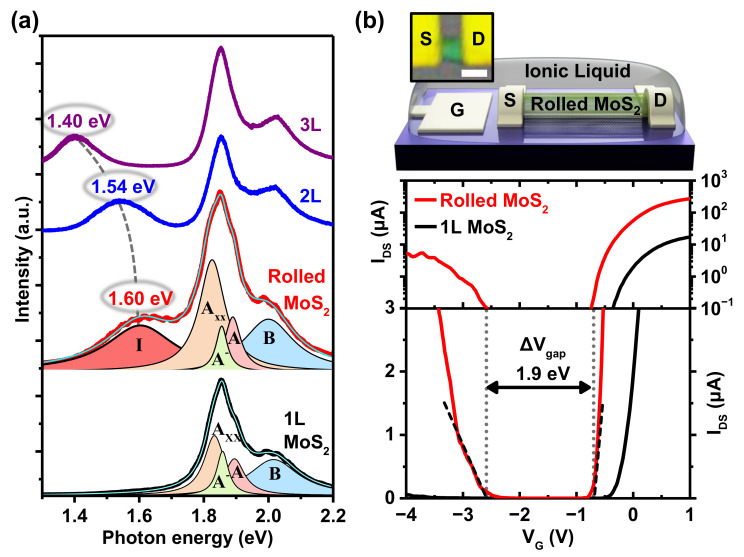
(**a**) PL spectrum of MoS_2_ nanoscroll (red) presented along with PL spectra of mono- (black), bi- (blue) and trilayer (purple) MoS_2_. Multipeak Lorentzian fittings are produced for the PL spectra of the scrolled MoS_2_ and MoS_2_ monolayer, and cyan solid lines represent fitted curves. (**b**) Schematic diagram of the ionic-liquid-gated transistor and an optical image of scrolled MoS_2_ transistor. White scale bar indicates 1 μm (top). Transfer characteristics of MoS_2_ nanoscroll and MoS_2_ monolayer transistors presented in semilogarithmic (middle) and in linear scales (bottom).

The optical and transport band gaps of scrolled MoS${}_{2}$ were determined from the PL spectroscopy and ionic-liquid gating of a FET device, respectively. Figure 2a presents the PL spectrum of the MoS${}_{2}$ nanoscroll shown in Figure 1a measured at Spot P2 with the PL spectra of mono-, bi-, and trilayer MoS${}_{2}$ sheets for comparison. The multipeak Lorentzian fitting of the PL spectrum of monolayer MoS${}_{2}$ exhibited A${}_{xx}$ (biexciton, 1.83 eV), A${}^{-}$ (trion, 1.86 eV), A (1.89 eV), and B (2.01 eV) exciton peaks. A and B excitons are related to the direct gap transitions between the conduction and the spin-split valence band [26]. For the MoS${}_{2}$ nanoscroll, in addition to the A${}_{xx}$, A${}^{-}$, A and B exciton peaks, a peak also appeared at ∼1.60 eV that could be associated with the I peak from an exciton in the indirect gap. The direct band gap of monolayer MoS${}_{2}$ changes into an indirect band gap in few-layer MoS${}_{2}$, and as the number of layers increases, the indirect gap decreases due to the quantum confinement effect that is common in nanosystems [32]. In Figure 2a, the I peak is shown at 1.54 and 1.40 eV for bi- and trilayer MoS${}_{2}$, respectively. The I peak was influenced by the interlayer coupling strength, and blue-shifted when the interaction was weak, as reported for folded and twisted MoS${}_{2}$ [3,33]. Therefore, an I peak at ∼1.60 eV, observed for MoS${}_{2}$ nanoscrolls, implies a weaker interlayer interaction in rolled MoS${}_{2}$ compared to that in Bernal-stacked bilayer MoS${}_{2}$. For scrolled MoS${}_{2}$, S atoms of the top layer may sit randomly relative to the S atoms of the bottom layer, resulting in a stronger repulsion between S atoms and a larger interlayer distance, i.e., a weaker interlayer interaction. PL spectra were measured at eight spots on two different MoS${}_{2}$ nanoscrolls, and the indirect band gap was in the range of 1.60±0.05 eV. In addition, one can notice a relatively weaker A${}^{-}$ peak for the MoS${}_{2}$ scroll compared to the monolayer MoS${}_{2}$ sheet. The A${}^{-}$ (trion) peak, related to the binding of a free electron to the A exciton, is strong when excess charge is present due to doping. Impurities or defects in SiO${}_{2}$ substrate result in a strong A${}^{-}$ peak for monolayer MoS${}_{2}$ [34]. For the MoS${}_{2}$ nanoscroll, the A${}^{-}$ peak was weaker as a significant portion of the scroll is separated from the substrate.

To further investigate the band gap of scrolled MoS${}_{2}$, ionic-liquid-gated transistors were fabricated on MoS${}_{2}$ nanoscrolls and on a MoS${}_{2}$ monolayer for reference. Figure 2b displays the transfer curves for an MoS${}_{2}$ nanoscroll ($r_{\mathrm{in}}≃300$ nm, ∼3 stacked rolls) and MoS${}_{2}$ monolayer transistors, presented with semilogarithmic (middle) and linear scales (bottom). A schematic diagram and microscopic image of the ionic-liquid-gated transistor are given at the top of Figure 2b, where the channel length was 800 nm. Ionic liquid DEME-TFSI was used as the gate dielectric. Because of the extremely large capacitance (≃8 μF/cm^2^ [35]) of the electric double layer that had accumulated on the MoS${}_{2}$ surface, the transport gap could be directly extracted from the transfer characteristics [36]. For a MoS${}_{2}$ nanoscroll transistor, the dependence of source-drain current $I_{DS}$ (measured at a source-drain voltage $V_{DS}=1$ V) on the gate voltage $V_{G}$ shows ambipolar behavior, and the transistor is in the off state when the Fermi level is located in the band gap of the nanoscroll. We found the threshold voltages for electron and hole conduction at $V_{th}^{e}≃-0.7$ V and $V_{th}^{h}≃-2.6$ V, respectively, which yielded a transport gap $\Delta V_{\mathrm{gap}}=e\left(V_{th}^{e}-V_{th}^{h}\right)=1.9$ eV for the scrolled MoS${}_{2}$. The transport gap (1.9 eV) of the MoS${}_{2}$ nanoscroll was larger than the optical gap (1.6 eV) by 0.3 eV, reflecting the exciton binding energy. Owing to increased dielectric screening in the MoS${}_{2}$ nanoscroll, the exciton binding energy can be smaller than the value (0.45 eV) reported for a MoS${}_{2}$ monolayer on a SiO${}_{2}$ substrate [37].

In comparison, the transfer characteristics of the MoS${}_{2}$ monolayer did not show ambipolar behavior, which is consistent with previous studies [38,39,40]. Sulfur vacancies induce hole-trap states inside the band gap of the MoS${}_{2}$ monolayer, 300–400 meV above the top of the valence band, and prevent hole conduction in exfoliated MoS${}_{2}$ monolayers. For few-layer MoS${}_{2}$, the defect states are located deep in the valence band, and it is possible to observe ambipolar transport [38,40]. The transport gap (∼1.9 eV) of the MoS${}_{2}$ nanoscroll, determined from Figure 2b, was larger than the transport gap (∼1.6 eV [38]) of the MoS${}_{2}$ bilayers, and smaller than the band gap (2.36–2.71 eV [41,42]) of the MoS${}_{2}$ monolayers. The ambipolar transport observed for the MoS${}_{2}$ nanoscroll implies that defect states due to sulfur vacancies are located inside the valence band.

### 3.3. Back-Gated Field Effect Transistor Based on Scrolled MoS${}_{2}$

**Figure 3:**
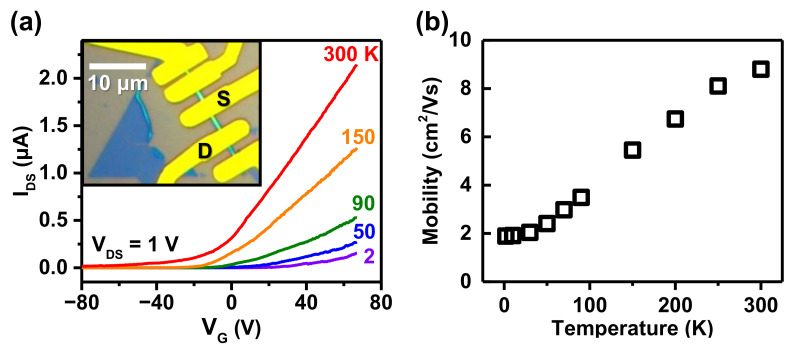
(**a**) Transfer characteristics of a MoS${}_{2}$ nanoscroll FET, measured at different temperatures between 2 and 300 K. Inset presents an optical image of the transistor. (**b**) Estimated field-effect electron mobility of the MoS${}_{2}$ nanoscroll as a function of temperature.

We also studied MoS${}_{2}$ nanoscrolls in a typical back-gated FET device fabricated on SiO${}_{2}$/Si substrate with source and drain electrodes (Ti/Au) patterned on top of scrolls. The inset of Figure 3a presents an optical image of a MoS${}_{2}$ nanoscroll FET, where electrodes were deposited on the rolled MoS${}_{2}$ shown in Figure 1a. Figure 3a shows the transfer curves of the device measured at different temperatures between 2 and 300 K. When the backgate voltage varied in the range of −80 ≤ $V_{G}$ ≤ 70 V, only n-type transport was observed for the nanoscroll FET, reflecting a much smaller back-gate capacitance (∼10 nF/cm${}^{2}$) compared with the ionic liquid gating. The source-drain current increased with increasing temperature, indicating the semiconducting nature of scrolled MoS${}_{2}$.

In Figure 3b, the field effect mobility, estimated for electron transport according to the 1D mobility equation $\mu_{1D}=\left(\Delta G/\Delta V_{G}\right)\cdot \left(L^{2}/C_{1D}\right)$, is displayed as a function of temperature. Here, *G* is the conductance, and the channel length is *L*≃ 3 μm. The capacitance is given by $C_{1D}=\frac{2\pi \epsilon \epsilon_{0}L}{{\cos h}^{-1}\left[\left(r_{\mathrm{out}}+t_{\mathrm{ox}}\right)/r_{\mathrm{out}}\right]}$, where ϵ(3.9) is the dielectric constant of SiO${}_{2}$ and $\epsilon_{0}$ is the permittivity of vacuum, with $t_{\mathrm{ox}}$ (300 nm) being the thickness of SiO${}_{2}$. For the MoS${}_{2}$ nanoscroll with $r_{\mathrm{out}}≃145$ nm, we have $C_{1D}≃3.7\times 10^{-16}$ F. The mobility at 300 K was estimated to be ∼8.8 cm${}^{2}$/Vs, an order of magnitude greater than the typical value of ∼1 cm${}^{2}$/Vs for our CVD-grown MoS${}_{2}$ monolayers. The enhancement of mobility of the MoS${}_{2}$ nanoscroll was first attributed to most of scroll’s surface being away from the substrate. Layers lifted from the substrate could avoid charge traps and be free from the influence of the substrate roughness and surface polar phonon scattering. In addition, since the outer layer was directly connected to the inner layer, all layers within the scroll could be available as current channels, unlike in multilayered 2D materials with high interlayer resistance. On the other hand, $\mu_{1D}$, estimated from the highest transconductance in the transfer curve, increased with *T*, as shown in Figure 3b, while the mobility of the MoS${}_{2}$ monolayer in a metallic conduction regime decreases with *T*, as mobility is limited by phonons at high temperatures [43]. However, the mobility of the MoS${}_{2}$ monolayer, when extracted in an insulating regime at lower gate voltages that locate the Fermi energy inside the band gap, showed a much lower value and increased with temperature [43]. Therefore, the transfer curves in Figure 3a are in the insulating regime with the Fermi energy inside the band gap for the range of $V_{G}$ applied, and the mobility was underestimated, particularly for lower temperatures. Scrolled MoS${}_{2}$ can be less doped, as most of the scroll’s surface is away from the substrate and affected less by charged impurities on the substrate.

### 3.4. Raman Spectra of Scrolled MoS${}_{2}$

**Figure 4:**
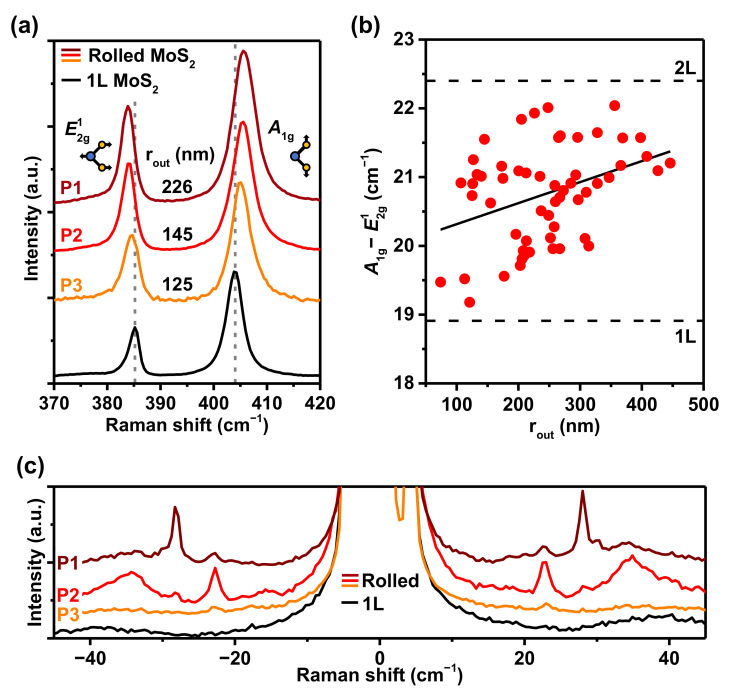
(**a**) Raman spectra of the MoS${}_{2}$ nanoscroll measured at Spots P1, P2, and P3 indicated in Figure 1a. $r_{\mathrm{out}}$ estimated for each spot is indicated. The Raman spectrum of MoS${}_{2}$ monolayer is also shown for reference. (**b**) Frequency difference between $E_{2g}^{1}$ and $A_{1g}$ modes as a function of $r_{\mathrm{out}}$ estimated for 56 different spots on 15 different MoS${}_{2}$ nanoscrolls. The solid line is the fitted slope. (**c**) Low-frequency (≤45 cm${}^{-1}$) Raman spectra of MoS${}_{2}$ monolayer and the MoS${}_{2}$ nanoscroll, measured at Spots P1, P2 and P3.

Figure 4a displays Raman spectra of the MoS${}_{2}$ nanoscroll shown in Figure 1a measured at Spots P1, P2, and P3, together with the Raman spectrum of a MoS${}_{2}$ monolayer as a reference. Raman-active $E_{2g}^{1}$ and $A_{1g}$ modes are observed in both monolayer and rolled MoS${}_{2}$. Compared to the $E_{2g}^{1}$ (∼405 ${\mathrm{cm}}^{-1}$) and $A_{1g}$ (∼385 ${\mathrm{cm}}^{-1}$) peaks of the MoS${}_{2}$ monolayer, $E_{2g}^{1}$ was red-shifted, and $A_{1g}$ is blue-shifted for the MoS${}_{2}$ nanoscroll, leading to a larger frequency difference between the two modes. This behavior is consistent with previous Raman studies on MoS${}_{2}$ nanoscrolls [10,13,14,16]. Raman spectroscopy performed on 56 different spots from 15 different MoS${}_{2}$ nanoscrolls resulted in average central positions of $E_{2g}^{1}$ and $A_{1g}$ modes at 383.6±0.4 and 404.4±0.6 cm${}^{-1}$, respectively, rendering the average frequency difference between the two modes to be 20.8±0.7 cm${}^{-1}$. The frequency distance between these modes is widely used to identify the number of layers in thin MoS${}_{2}$, as the distance increases with the number of layers from ∼18.8 (mono), 22.4 (bi) to 24.8 cm${}^{-1}$ (bulk) [44].

The value (∼20.8 cm${}^{-1}$) observed for MoS${}_{2}$ nanoscrolls was between those of the MoS${}_{2}$ monolayer and bilayer. Considering that the average number of concentric layers in our nanoscrolls was ∼4, the observed distance between the two modes indicates a weaker interlayer interaction for the MoS${}_{2}$ nanoscroll compared to the few Bernal-stacked MoS${}_{2}$ layers, which is consistent with our PL results in Figure 2a. In folded or twisted MoS${}_{2}$, a narrower distance between the two modes than that for the same layer number of Bernal-stacked MoS${}_{2}$ was reported and attributed to the weaker interlayer interactions [3,45].

The frequency difference between the $E_{2g}^{1}$ and $A_{1g}$ modes for all 56 measurements is plotted in Figure 4b as a function of $r_{\mathrm{out}}$. No clear dependence was observed for the height/width ratio or the number of stacking layers in the nanoscroll; the distance between the two peaks tended to increase with increasing $r_{\mathrm{out}}$. The increase in $r_{\mathrm{out}}$ led to a decrease in strain due to the reduced curvature that, however, would be expected to show the opposite dependence. Suspended MoS${}_{2}$ monolayers under strain show a decrease in peak separation as the strain is reduced [46]. Instead, a larger $r_{\mathrm{out}}$ may facilitate some parts of the layers to be Bernal-stacked, giving rise to a stronger interlayer interaction and wider distance between the two Raman peaks.

Figure 4c shows the ultralow-frequency (≤45 cm${}^{-1}$) Raman spectra of the MoS${}_{2}$ monolayer and the MoS${}_{2}$ nanoscroll measured at Spots P1, P2 and P3. For the MoS${}_{2}$ monolayer, there was no peak in the frequency range. On the other hand, for the MoS${}_{2}$ nanoscroll (P1 spot), a sharp peak was observed at ∼28 cm${}^{-1}$ with the full width at half maximum (FWHM) of ∼1 cm${}^{-1}$, and small peaks were located at 22.5, 34.5 cm${}^{-1}$. For Spot P2, there was a sharp peak at ∼22.5 and a broad peak at 34.5 cm${}^{-1}$ with FWHM of ∼1 and 10 cm${}^{-1}$, respectively; for Spot P3, only a weak peak was seen at ∼22.5 cm${}^{-1}$. For multilayer MoS${}_{2}$, shear (C mode) and layer breathing modes (LB mode) were observed in this low frequency range [47]. For MoS${}_{2}$ nanoscrolls, LB modes at 21, 26, 38 cm${}^{-1}$ were reported [16]. Our results indicate that there can be LB or C mode coupling between the concentric layers of MoS${}_{2}$ nanoscrolls. These modes are sensitive to the number of layers and stacking configurations [45]; therefore, the peaks appear at different frequencies for different spots depending on the detailed configuration and topology of the nanoscroll.

## 4. Conclusions

In summary, we formed MoS${}_{2}$ nanoscrolls from both CVD-grown and exfoliated MoS${}_{2}$ monolayers that had inner core radii of ∼250 nm, and investigated their optical and transport band gaps. PL spectroscopy reveals that the MoS${}_{2}$ monolayer, originally a direct band-gap semiconductor (∼1.85 eV (optical)), changed into an indirect band-gap semiconductor (∼1.6 eV) upon scrolling. The size of the indirect gap for MoS${}_{2}$ nanoscrolls was larger than that of the MoS${}_{2}$ bilayer (∼1.54 eV), implying a weaker interlayer interaction between the concentric layers in the nanoscroll compared to that in few Bernal-stacked MoS${}_{2}$ layers. We also determined the transport band gap (∼1.9 eV) of MoS${}_{2}$ nanoscrolls by fabricating and characterizing ambipolar ionic-liquid-gated transistors. The difference between the transport and optical gaps suggests an exciton binding energy of 0.3 eV for MoS${}_{2}$ nanoscrolls. Rolling up 2D atomic layers into nanoscrolls introduces a new type of quasi-1D nanostructure and provides another way to tune the band gap of 2D materials.

## Data Availability

Not applicable.
